# Cardiac tamponade revealing tuberculous pericarditis: a case report

**DOI:** 10.1099/acmi.0.000983.v4

**Published:** 2025-05-02

**Authors:** Oumaima Skalante, Mariam Hachimi Idrissi, Soukaina Cherkaoui, Elmostafa Benaissa, Yassine Ben Lahlou, Mariama Chadli

**Affiliations:** 1 Department of Bacteriology, Mohammed V Military Teaching Hospital, Rabat, Morocco; 2 Department of Cardiology, Mohammed V Military Teaching Hospital, Rabat, Morocco

**Keywords:** cardiac tamponade, GenXpert MTB/RIF Ultra, pericarditis, tuberculosis

## Abstract

Tuberculosis remains a major public health issue in Morocco. Pulmonary tuberculosis is the most common form, but various extrapulmonary forms exist. Tuberculous pericarditis is a rare form of extrapulmonary tuberculosis that can be complicated by cardiac tamponade, pericardial constriction or their combination, which can threaten the patient’s life. Its clinical and radiological signs are nonspecific, and the clinical presentation can be misleading and incomplete, sometimes even with an initial tamponade. We report the case of a 68-year-old female patient admitted for intense retrosternal chest pain associated with acute dyspnoea, evolving in the context of unquantified weight loss and general deterioration. Additionally, she reported a history of fever and night sweats. Clinical examination revealed a conscious, febrile, hypotensive, tachycardic, polypneic patient with good oxygen saturation, signs of right heart failure and muffled heart sounds on auscultation. Chest X-ray revealed cardiomegaly, and the ECG showed diffuse low voltage. Given the presence of Beck’s triad suggestive of cardiac tamponade, a transthoracic echocardiogram was performed, revealing a large pericardial effusion with a ‘swinging heart’. A chest CT scan also confirmed the large pericardial effusion. The diagnosis of cardiac tamponade was made based on the clinical and radiological findings, and pericardial drainage was performed, after which the patient showed clinical improvement. PCR GenXpert MTB/RIF Ultra detected the presence of *Mycobacterium tuberculosis* in the pericardial fluid, with no resistance to rifampicin. Culture was positive for *M. tuberculosis*. The diagnosis of tuberculous pericarditis was, thus, confirmed, and the patient was started on quadruple antituberculosis therapy with good clinical progress.

## Data Summary

No data were generated during this research or are required for the work to be reproduced.

## Introduction

Tuberculosis remains a major public health issue in Morocco [1]. Pulmonary tuberculosis is the most common form, but various extrapulmonary forms also exist, including pericardial tuberculosis, which is rare and accounts for 1–2% of tuberculosis infections [2]. This pericardial form can be life-threatening due to its complications such as cardiac tamponade and constriction, or their combination. We report the case of a patient who presented with cardiac tamponade due to tuberculous pericarditis.

## Case presentation

A 68-year-old immunocompetent patient with a history of grade I arterial hypertension under treatment and no other significant medical history was admitted for acute thoracic pain relieved by anteflexion with acute dyspnoea.

The onset of her illness occurred a few days before admission, with the development of acute retrosternal pain associated with New York Heart Association stage IV dyspnoea, all in the context of weight loss and a deterioration of her general condition. The patient reported fever and night sweats. These symptoms persisted despite symptomatic analgesic and antipyretic treatment.

Clinical examination revealed a conscious, febrile patient with a blood pressure of 78/40 mmHg, tachycardia at 106 beats min^−1^ and signs of right heart failure: jugular vein distension and positive Kussmaul’s sign. On cardiac auscultation, heart sounds were muffled with no pericardial rub or murmurs in the large accessible vascular axes. Pulmonary examination revealed polypnoea at 27 cycles min^−1^ and room air SaO₂ of 98%. The rest of the clinical examination was unremarkable.

**Fig. 1.**
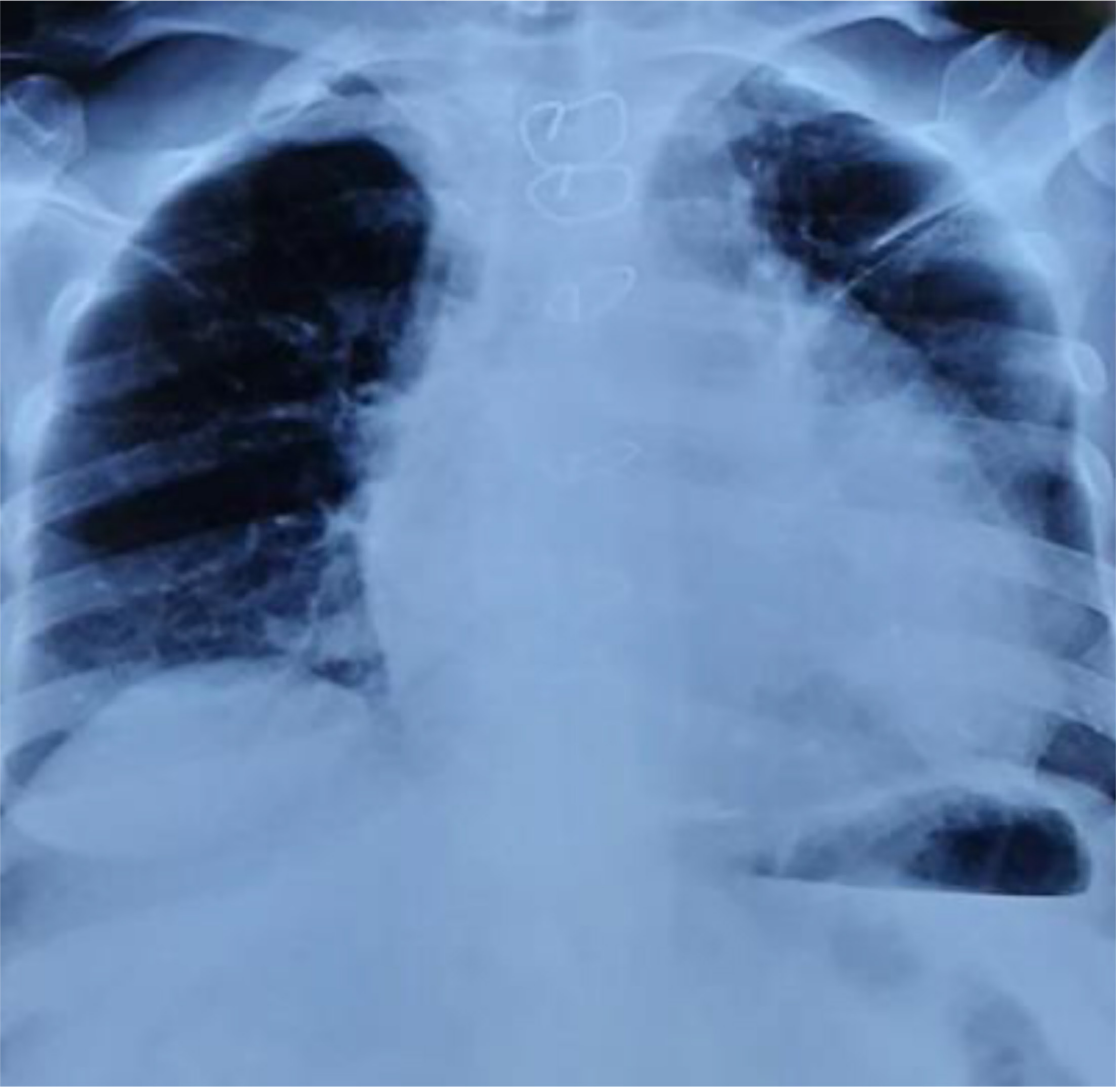
Chest X-ray showing the cardiomegaly.

Chest X-ray revealed cardiomegaly (Fig. 1), and the electrocardiogram (ECG) showed diffuse microvoltage.

**Fig. 2.**
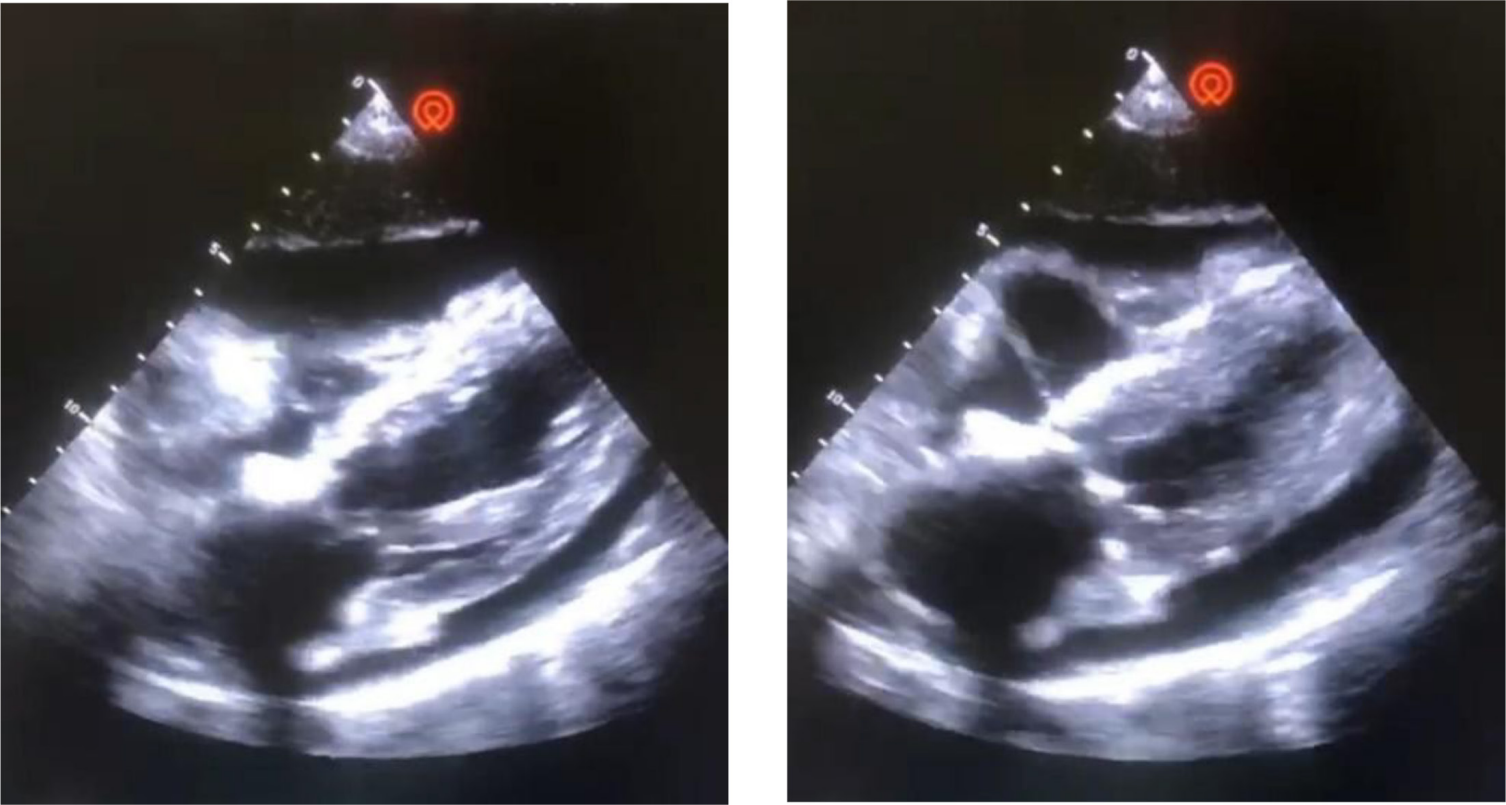
TTE showing the large pericardial effusion with swinging heart and collapse of the right atrium.

Given this clinical and radiological picture suggestive of cardiac tamponade, a transthoracic echocardiography (TTE) was performed, revealing a large circumferential pericardial effusion with swinging of the heart and collapse of the right atrium in end diastole (Fig. 2).

**Fig. 3.**
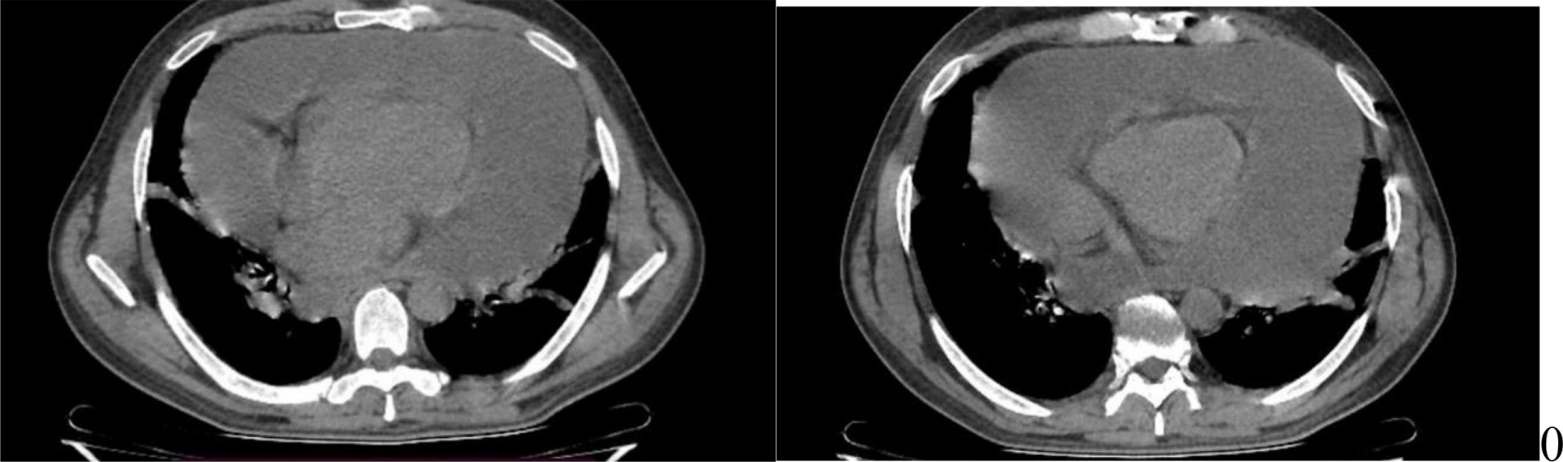
Thoracic scan: revealing the abundant pericardial effusion.

The thoracic Computed tomography (CT) scan revealed a circumferential pericardial effusion of great abundance, with no other associated involvement (Fig. 3).

Biological tests revealed an inflammatory syndrome, with a white blood cell count of 10,200 mm^−^³, a C-reactive protein level of 48 mg l^−1^ and troponin at 13 ng l^−1^. The patient was Human immunodeficiency virus (HIV)-negative.

The diagnosis of cardiac tamponade was established in view of the patient’s clinical and radiological signs, and the indication for therapeutic and diagnostic pericardial drainage was made. Following this, the patient showed clinical improvement, with regression of the pericardial effusion on TTE. Pericardial puncture yielded 800 ml of citrine-yellow fluid.

The punctured pericardial fluid was sent to the bacteriology laboratory. After centrifugation, the pellet was decontaminated with Lauryl sulphate solution, and then it was neutralized. From this pellet, a smear was taken, stained with Ziehl–Neelsen stain and read under the microscope with objective 1,000, but without any Bacillus acido-alcohol resistant (BAAR) visualized.

**Fig. 4.**
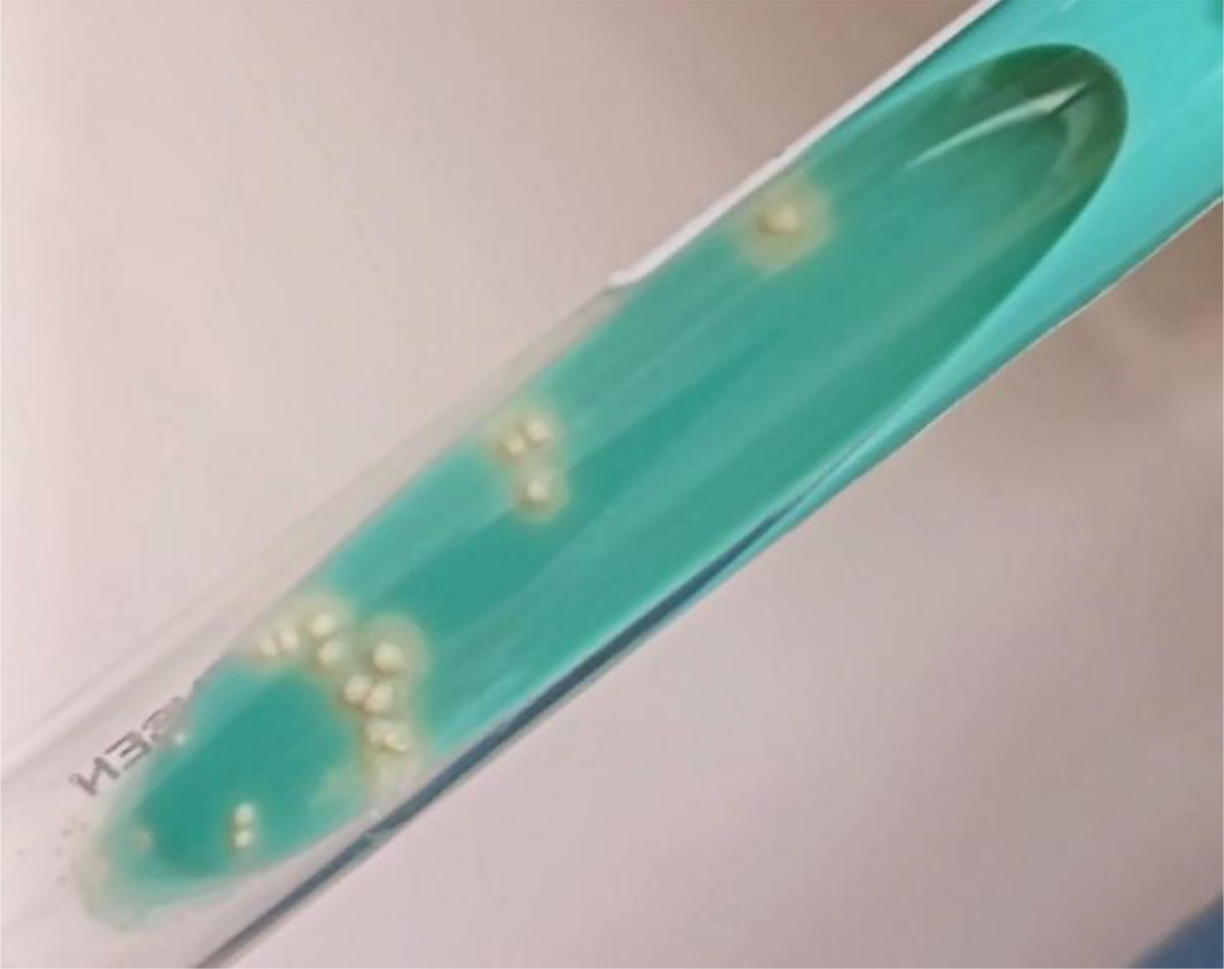
Culture images showing *Mycobacterium tuberculosis* colonies on LJ solid medium.

A total of 200 μl of the pellet was then inoculated onto Lowenstein–Jensen (LJ) solid medium. The tubes were incubated at 37 °C, half-open and in a tilted position. This culture was positive after 31 days, yielding 15 creamy-beige, dry, rough-surfaced, cauliflower-like colonies (Fig. 4).

A GeneXpert MTB/RIF Ultra test was performed: 1 ml of the centrifuged pericardial fluid pellet was transferred into a conical tube containing 2 ml of sample reagent, and the mixture was vortexed for 10 s. After a 10 min pause, the sample was aspirated up to the mark on the provided transfer pipette and transferred into the sample chamber of the Xpert MTB/RIF Ultra cartridge. The results were as follows: *Mycobacterium tuberculosis* (MTB) was detected at low levels, and Rifampicin resistance was not detected.

The standard culture was sterile after 48 h of incubation.

Biochemical analysis of this pericardial fluid revealed an exudative fluid with a protein level of 53 g l^−1^ and an Lactate dehydrogenase (LDH) of 2654 U l^−1^.

To identify any pulmonary involvement of tuberculosis, the patient’s sputum was tested for tuberculosis over three consecutive days. Direct examination of the sputum after Ziehl–Neelsen staining was negative, and cultures on LJ solid medium remained sterile after 2 months of incubation. Chest X-rays showed no abnormalities suggestive of pulmonary tuberculosis or sequelae.

In summary, the diagnosis of cardiac tamponade due to tuberculous pericarditis was established. The patient underwent pericardiocentesis and was started on a four-drug regimen in accordance with the National Tuberculosis Treatment Guidelines (2RHZE/4RH): 2 months of Rifampicin, Isoniazid, Ethambutol and Pyrazinamide, followed by 4 months of Rifampicin and Isoniazid, in combination with corticosteroid therapy. The patient responded well to treatment, and her condition improved significantly.

## Discussion

Pericarditis is the inflammation of both layers of the pericardium, clinically characterized by typical chest pain, sometimes accompanied by pericardial effusion [3]. This inflammation may be due to infections: viral, especially in developed countries; tuberculosis, particularly in developing countries; and rarely fungal; or non-infectious causes: autoimmune, neoplastic, traumatic or others [4,5].

Tuberculosis, a public health problem in Morocco, is one of the causes of pericarditis. It primarily affects the lungs, with extrapulmonary forms representing 48% of tuberculosis cases [1].

It causes subacute pericarditis, associated with a general deterioration in condition and persistent moderate fever [6], and is characterized by abundant, often circumferential pericardial effusion, with a risk of cardiac tamponade and mortality. The clinical picture can be misleading and incomplete or may even present with an inaugural tamponade [7].

Cardiac tamponade, a complication of pericarditis, is manifested by the Beck triad, which includes arterial hypotension, distension of the neck veins and muffled heart sounds [8]. Other complications of tuberculous pericarditis may include recurrence or even pericardial constriction [6]. In our patient’s case, the clinical presentation was similar to that reported in the literature, with the presence of Beck’s triad upon admission.

Given the cardiac tamponade in our patient, pericardial fluid drainage was indicated, and its analysis was performed according to the European Society of Cardiology (ESC) and French Society of Cardiology (SFC) guidelines. The results were as follows: biochemical analysis revealed an exudative fluid, and cytological examination showed abundant cellularity consisting of red blood cells and leukocytes in fresh direct examination, with no bacterial flora observed in the Gram stain and no detection of MTB in the Ziehl–Neelsen stain. The standard culture was sterile, whereas the mycobacterial culture was positive after 1 month of incubation. PCR was performed using the GeneXpert MTB/RIF Ultra test, which detected a low-level MTB complex without resistance to Rifampicin [5,6].

The Genexpert MTB/RIF Ultra test is primarily approved by the manufacturer for sputum samples to detect pulmonary tuberculosis [9]. However, numerous studies have highlighted its utility in diagnosing extrapulmonary tuberculosis, including pericardial tuberculosis, despite its official approval being limited to pulmonary samples [10,11]. These findings suggest that the test could serve as a valuable diagnostic tool for pericardial tuberculosis, though additional validation is required before it can be widely adopted.

The sensitivity of this test for extrapulmonary samples is particularly relevant in individuals with paucibacillary tuberculosis and extrapulmonary tuberculosis. According to the study by Piersimauni *et al*., it was 95% in individuals with extrapulmonary tuberculosis who had a negative smear but a positive culture [12].

According to another study conducted in the same context to analyse its effectiveness in detecting MTB DNA, its sensitivity is 75.9% in extrapulmonary samples with a negative smear and positive culture, and its specificity is 100% [13].

This is consistent with the results of our patient, in whom this test quickly revealed the presence of the MTB complex, despite the negative smear. Furthermore, its rapidity helps reduce the diagnostic delay in patients, without the need to wait for the results of the culture, which is relatively slow and could delay therapeutic management.

## Conclusion

GenXpert MTB/RIF Ultra has significantly advanced the diagnosis of extrapulmonary forms of tuberculosis, including pericardial involvement, due to its high sensitivity, specificity and rapid results.

This enables early and appropriate management, especially in countries where tuberculosis is endemic, and can be the cause of pericarditis, with pericardial effusion whose clinical and radiological signs may be non-specific, and which, in some cases, may initially manifest as life-threatening cardiac tamponade.

## Abbreviations

BAAR: bacillus acido-alcohol resistant
CT: computed tomography
ECG: electrocardiogram
ESC: European Society of Cardiology
HIV: human immunodeficiency virus
LDH: lactate dehydrogenase
LJ: Lowenstein–Jensen
MTB: *Mycobacterium tuberculosis*
SFC: French Society of Cardiology
TTE: transthoracic echocardiography
